# Physiology and Multi-Omics Provide Insights into Sperm Activation and Movement in Euryhaline Spotted Seabass (*Lateolabrax maculatus*)

**DOI:** 10.3390/biom16071021

**Published:** 2026-07-13

**Authors:** Qinghua Wang, Yuxin Zhang, Weiwei Zhang, Yingxin Wu, Jiajie Li, Yizheng Zhang, Lu Li, Zhiming Zhu, Zining Meng

**Affiliations:** 1 School of Life Sciences, State Key Laboratory of Biocontrol/Guangdong Core Germplasm Bank for Marine Economic Animals, Southern Marine Science and Engineering Guangdong Laboratory (Zhuhai)/Guangdong Provincial Key Laboratory of Aquatic Economic Animals, Sun Yat-Sen University, Guangzhou 510275, China; wangqh55@mail2.sysu.edu.cn (Q.W.); zhangyx558@mail2.sysu.edu.cn (Y.Z.); zhangww79@mail2.sysu.edu.cn (W.Z.); wuyx328@mail2.sysu.edu.cn (Y.W.); lijj379@mail2.sysu.edu.cn (J.L.); zhangyzh253@mail2.sysu.edu.cn (Y.Z.); lilu95@mail2.sysu.edu.cn (L.L.); 2 China-ASEAN Belt and Road Joint Laboratory on Mariculture Technology, Guangzhou 510275, China

**Keywords:** *Lateolabrax maculatus*, euryhaline fishes, physiology, transcriptomics and proteomics, osmotic regulation and ionic signaling, sperm activation and movement mechanisms

## Abstract

Sperm activation and movement are pivotal determinants of fertilization success in teleosts, yet the molecular mechanisms in euryhaline species remain largely unresolved. Here, we integrated physiology and multi-omics to elucidate the osmolality, ions, and regulatory networks underlying sperm activation and movement in spotted seabass (*Lateolabrax maculatus*). Spotted seabass sperm showed strong motility across a broad osmotic range of 500 to 1200 mOsm/kg, with Na^+^ and K^+^ promoting motility and Ca^2+^ exerting inhibitory effects. Integrated transcriptomics and proteomics identified 29 genes and 6 proteins as candidate molecules associated with Na^+^ and K^+^ signaling, Ca^2+^ signaling and apoptosis, and energy metabolism. Up-regulation of NHE1 suggested a potential involvement of Na^+^/H^+^ exchange that regulated Na^+^ influx, while K^+^ influx via activated K^+^ channels may depolarize membrane potential. The *capn1* was up-regulated, which may be associated with Ca^2+^-mediated apoptosis. Euryhaline fishes exhibited a broader osmotic activation range than freshwater and marine species, underscoring the critical roles of osmotic adaptability in fish sperm physiology. Ionic regulation patterns of euryhaline fishes are generally consistent with their environmental adaptation, with seawater-adapted species resembling marine fishes and freshwater-adapted species resembling freshwater fishes. Our findings highlight the integrated roles of osmotic effects and ionic regulation, providing insights into sperm activation and movement in euryhaline spotted seabass.

## 1. Introduction

In teleosts, spermatozoa remain quiescent or inactive in the vas deferens prior to spawning and become activated upon release into the aquatic environment, where shifts in osmolality, ions, and energy serve as key activators [1,2]. Low osmolarity stimulates activation in freshwater species, whereas hyperosmotic seawater acts as a trigger for sperm motility in marine fishes [3,4]. Compared with freshwater species, marine fish spermatozoa typically sustain motility for a longer duration post-activation, a difference attributable to their higher ATP and energy reserves [3,4]. Ion types and concentrations exhibit divergent effects on sperm motility across teleost fish. Na^+^ generally exerts a stimulatory effect in both marine and freshwater environments [5,6]. In contrast, K^+^ exhibits dual effects, inhibiting sperm motility in some freshwater fishes while contributing to membrane depolarization and motility initiation in marine species [7,8]. Sperm activation occurs independently of extracellular Ca^2+^ in several teleosts, including catfish (*Clarias batrachus*) [9], zebrafish (*Danio rerio*) [10], and rainbow trout (*Oncorhynchus mykiss*) [11], whereas in species such as common carp (*Cyprinus carpio* L.) [12] and marbled goby (*Oxyeleotris marmorata*) [9], extracellular Ca^2+^ is essential for motility initiation. It follows that ion-dependent regulation exhibits pronounced species specificity. Euryhaline teleosts display sperm activation across a broad salinity spectrum, indicative of strong physiological adaptability. However, compared with marine and freshwater fishes, the molecular mechanisms underlying sperm activation and movement in euryhaline species remain insufficiently understood.

Advances in high-throughput sequencing technologies have facilitated the integration of transcriptomic and proteomic analyses [13,14], providing a powerful framework to characterize gene and protein functions and elucidate the molecular mechanisms underlying sperm activation and movement in fish. Transcriptomic analysis reveals gene expression patterns in gilthead seabream (*Sparus aurata*) during spermiogenesis, with significant enrichment in pathways associated with signaling, metabolism, ion transport, and cytoskeletal dynamics [14]. Meta-analyses have further identified the expression of ion channel-related genes, particularly those related to K^+^ and Ca^2+^ transport, highlighting the important roles of ion-dependent regulatory mechanisms in fish sperm physiology [15]. Proteomic studies show that fish spermatozoa contain a broad range of proteins associated with protein turnover, signal transduction, intracellular transport, cytoskeletal organization, and stress responses, as well as metabolic pathways such as glycolysis, lipid metabolism, and the tricarboxylic acid (TCA) cycle that sustain sperm function and motility [16,17,18,19]. Moreover, a subset of proteins in spermatozoa is associated with specialized functions, including immune responses, detoxification, and adaptation to environmental conditions [20]. These findings highlight species-specific variations in sperm activation and underscore the potential of integrated omics approaches to resolve the molecular regulatory networks of sperm activation and movement in euryhaline species.

Spotted seabass (*Lateolabrax maculatus*), a typical euryhaline teleost, exhibits remarkable adaptability, surviving across broad salinities from 0 to 38‰ [21,22]. Widely distributed along the coasts of China, Japan, and the Korean Peninsula [23], it is both ecologically significant and one of the four major marine aquaculture fish species in China, with production exceeding 240,000 tons in 2023 [24]. In this study, we integrated sperm motility analysis with transcriptomic and proteomic profiling to dissect the regulatory mechanisms of sperm activation and movement in this euryhaline species. Our physiological analysis coupled with multi-omics data revealed the influence of osmotic and ionic factors and identified critical genes, proteins, and molecular regulatory networks. These findings provide a mechanistic framework for understanding sperm activation and movement in euryhaline teleosts, offering vital insights for improving fertilization and aquaculture breeding success.

## 2. Materials and Methods

### 2.1. Ethics Statement, Broodstock Maintenance, and Sperm Collection

**Figure 1:**
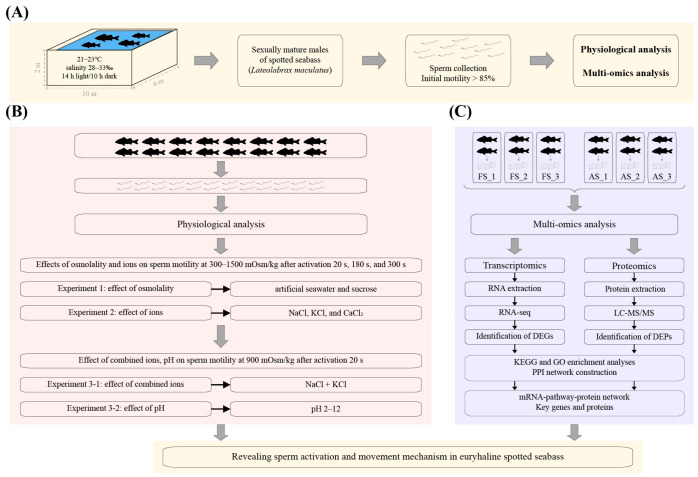
Technical framework. (**A**) Broodstock maintenance and sperm collection, (**B**) physiological analysis, and (**C**) transcriptomics and proteomics analyses.

All experimental procedures were approved by the Institutional Animal Care and Use Committee of the School of Life Sciences, Sun Yat-sen University (No. SYSU-LS-IACUC-2024-0146). Experiments were performed during the reproductive season of spotted seabass from October 2024 to November 2025. Sexually mature males (body weight > 2.5 kg, age > 3 years) were obtained from Zhiqiang Aquafarm (Zhangzhou, Fujian, China). Broodstock were maintained indoors in a 10 m × 6 m × 2 m concrete tank supplied with aerated flow-through seawater (salinity 28–33‰, temperature 21–23 °C) under a natural photoperiod (14 h light, 10 h dark). Approximately 50% of seawater was renewed daily. Following eugenol anesthesia (5–10 min), sperm were collected from sexually mature males by abdominal massage and stored in 15 mL RNase-free Eppendorf tubes at 4 °C. Motility was assessed using Integrated Semen Analysis Systems (ISAS v2.0, Spain), and only uncontaminated samples (without blood, urine, or feces) with >85% initial motility were used for physiological assessment as well as transcriptomics and proteomics analyses (Figure 1A).

### 2.2. Physiological Analysis

A total of 16 males were used for physiological analysis. Sperm samples from two fish were pooled and used to determine the effects of osmolality and ionic composition on sperm motility after activation (Figure 1B). Each treatment was conducted in at least three replicates. For motility analysis, 0.5 μL of sperm was mixed with 40 μL of activation solution and gently pipetted to ensure activation. The suspension was immediately observed under an optical microscope equipped with a color CCD camera (Olympus Corporation, Tokyo, Japan). Sperm motility was classified into four levels by the ISAS v.2.0, including grade A (rapid progressive spermatozoa), grade B (slow progressive spermatozoa), grade C (non-progressive spermatozoa showing active movement without obvious forward progression), and grade D (immotile spermatozoa). Total sperm motility was calculated as the sum of grades A, B, and C. The corresponding classification criteria were based on the default settings of the ISAS v.2.0 sperm analysis system. Motility parameters were quantified using the ISAS v.2.0 after sperm activation at 20 s, 180 s, and 300 s, representing the early activation stage, motility maintenance, and late-stage decline, respectively. Using filtered seawater (926 ± 19 mOsm/kg) as the activation medium, motility decreased progressively from 88.69 ± 3.23% at activation 20 s to 52.82 ± 4.28% at 180 s and 33.46 ± 3.15% at 300 s. Activation solutions were prepared from sucrose, artificial seawater (mainly containing Na^+^, Mg^2+^, Ca^2+^, K^+^, and Sr^2+^), NaCl, KCl, and CaCl_2_ (Yier, Guangzhou, China). All reagents were dissolved in distilled water, and the pH was adjusted to 7.8–8.2 using an FE20K Plus pH meter (Mettler Toledo, Shanghai, China). Preliminary experiments indicated that sperm can be activated across an osmotic range of 400–1400 mOsm/kg, and thus stock solutions at 2000 mOsm/kg were prepared to generate activation solutions ranging from 300 to 1500 mOsm/kg at 100 mOsm/kg intervals. The 2000 mOsm/kg stock solutions contained 90 g/L (1406 mM) artificial sea salt, 2000 mM sucrose, 1000 mM NaCl, 1000 mM KCl, and 666 mM CaCl_2_, respectively. Sperm samples were centrifuged twice at 12,000× *g* for 5 min at 4 °C to isolate seminal plasma. The osmolality of seminal plasma from spotted seabass was measured using an Osmomat 030 cryoscopic osmometer (Gonotec GmbH, Berlin, Germany) and ranged from 316 to 322 mOsm/kg.

#### 2.2.1. Experiment 1: Effect of Osmolality on Sperm Motility

The influence of osmolality on sperm motility was examined by activating sperm samples in sucrose and artificial seawater ranging from 300 to 1500 mOsm/kg, with motility measured after sperm activation at 20 s, 180 s, and 300 s. Sucrose solutions were employed to evaluate osmotic effects in the absence of ions, while artificial seawater served as a multi-ionic reference medium.

#### 2.2.2. Experiment 2: Effect of Ions on Sperm Motility

To assess the effect of individual ions, sperm were activated in NaCl and KCl solutions within the same osmolality range, and motility was recorded at 20, 180, and 300 s post-activation.

Considering the specific properties of Ca^2+^, sperm in CaCl_2_ activation media were completely immotile after 180 s; thus, sperm motility was recorded at 20 and 60 s after activation.

#### 2.2.3. Experiment 3: Effect of Combined Ions and pH on Sperm Motility

Based on Experiments 1 and 2, 900 mOsm/kg was identified as the optimal osmolality for sperm activation. To investigate the combined effects of Na^+^ and K^+^, sperm samples were activated at molar ratios of 7:1, 5:1, 3:1, 1:1, 1:3, 1:5, and 1:7 under 900 mOsm/kg, and motility was evaluated at 20 s post-activation.

### 2.3. Transcriptomics Analysis

#### 2.3.1. Preparation of Omics Samples

A total of 12 males were used for multi-omics analysis. Sperm samples from six males were activated with naturally filtered seawater for 600 s to generate the activated sperm (AS) group. Each individual fish was treated as an independent biological replicate. Three biological replicates were used for transcriptomic profiling, and the remaining three were used for proteomic analysis. At this time point, sperm had been fully activated, facilitating more complete acquisition of transcripts and proteins. This process encompassed early activation, motility maintenance, and motility decline. The fresh sperm (FS) group from six males without activation served as the control. Sperm pellets were collected by centrifugation at 12,000× *g* for 5 min at 4 °C, immediately frozen in liquid nitrogen, and stored at −80 °C for subsequent library construction (Figure 1C).

#### 2.3.2. RNA Extraction, Transcriptomic Library Construction, and RNA-Seq

Total RNA was isolated from sperm using TRIzol reagent (Invitrogen, Carlsbad, CA, USA) according to the manufacturer’s instructions. RNA integrity and quality were assessed using an Agilent 2100 Bioanalyzer (Agilent Technologies, Palo Alto, CA, USA). Polyadenylated mRNA was enriched using oligo (dT) magnetic beads and fragmented into short fragments. First-strand cDNA was synthesized using random hexamer primers, followed by second-strand synthesis. The resulting cDNA fragments were purified, end-repaired, A-tailed, and ligated to adapters. The constructed libraries were sequenced on an Illumina NovaSeq 6000 platform (Illumina, San Diego, CA, USA) by Novogene Co., Ltd. (Beijing, China).

#### 2.3.3. Quality Control, Transcript Assembly, and Differential Expression Analysis

Raw reads were filtered with fastp [25] to remove adapters, poly-N sequences, and low-quality reads. Read quality was evaluated using Q20, Q30, and GC content. Clean reads were aligned to the *L. maculatus* reference genome (GCA_031216445.1) [26] using Hisat v.2.2.1 [27], transcripts were assembled with StringTie v.2.2.3 [28], and transcripts not annotated in the reference genome were classified as novel genes. Gene expression was quantified as fragments per kilobase of transcript per million mapped reads (FPKM) values using RSEM [29]. Differential expression analysis was performed using DESeq2 v.1.42.0 [30], and genes with a false discovery rate < 0.05 and |log2 (fold change)| > 1 were considered differentially expressed genes (DEGs).

### 2.4. Proteomics Analysis

#### 2.4.1. Total Protein Extraction, Protein Quality Assessment, and Trypsin Treatment

In this section, the preparation of omics samples was carried out as described in Section 2.3.1. Sperm samples were lysed in lysis buffer containing 6 M urea and 100 mM TEAB (pH 8.5), followed by ultrasonication on ice for 5 min. The lysates were centrifuged at 12,000× *g* for 15 min at 4 °C, and the supernatants were collected. Proteins were reduced with 1 M dithiothreitol (DTT; Sigma, MO, USA) at 56 °C for 1 h and subsequently alkylated with iodoacetamide for 1 h at room temperature in the dark. The reaction was quenched by incubation on ice for 2 min. Protein concentrations were determined using the Bradford assay (Beyotime, Shanghai, China) with bovine serum albumin (BSA; 0–0.5 μg/μL). Standard and diluted samples (20 μL) were added to 96-well plates in triplicate, followed by the addition of 180 μL of G250 dye reagent (Bio-Rad Laboratories, Inc., Hercules, CA, USA). After incubation at room temperature for 5 min, absorbance was measured at 595 nm, and protein concentrations were calculated from the BSA standard curve. Equal amounts of protein (20 μg) were separated by 12% SDS-PAGE (120 V for 20 min, then 150 V for 50 min) and visualized via Coomassie Brilliant Blue R-250 staining and destaining. Protein samples were diluted with lysis buffer to a final volume of 100 μL and digested with trypsin at 37 °C for 4 h. Digested samples were acidified with formic acid to pH < 3 and centrifuged at 12,000× *g* for 5 min at room temperature. The resulting supernatants were loaded onto C18 desalting columns, washed three times with 0.1% formic acid in 3% acetonitrile, and eluted with 0.1% formic acid in 70% acetonitrile. The eluates were collected and lyophilized prior to further analyses.

#### 2.4.2. Liquid Chromatography–Tandem Mass Spectrometry (LC-MS/MS) Analysis

Peptides were dissolved in 10 µL of solvent A (99.9% water, 0.1% formic acid), centrifuged at 14,000× *g* for 20 min at 4 °C, and 200 ng of the supernatant was injected for LC-MS analysis. Peptide separation was performed on a Vanquish Neo UHPLC system (Thermo Fisher Scientific, Waltham, MA, USA) using a C18 pre-column (174,500, 5 mm × 300 µm, 5 µm) and a C18 analytical column (ES906, PepMap™ Neo UHPLC, Thermo Fisher Scientific, Waltham, MA, USA, 150 µm × 15 cm, 2 µm), which was maintained at 50 °C. MS analysis was conducted on an Orbitrap Astral mass spectrometer (Thermo Fisher Scientific, Waltham, MA, USA) coupled with an Easy-spray (ESI) source operating in data-independent acquisition (DIA) mode. Full MS scans were acquired from *m*/*z* 380 to 980 at a resolution of 240,000 with an AGC target of 500% and 2 Th isolation windows across 300 DIA segments. MS2 spectra were acquired over an *m*/*z* range of 150–2000 at an Astral resolution of 80,000 with an NCE of 25% and a maximum injection time of 3 ms.

#### 2.4.3. Identification and Quantitation of Proteins

Raw mass spectrometry data were processed using DIA-NN against the *L. maculatus* protein database. Precursor and fragment ion mass errors were automatically detected and corrected. Carbamidomethylation of cysteine was set as a fixed modification, and N-terminal methionine excision was set as a variable modification, with up to two missed cleavages permitted. Peptide and protein identifications were filtered at Global Q-Value and PG Q-Value thresholds of 0.01 to ensure high confidence. Proteins with *p* < 0.05 and fold change (FC) > 1.2 or <0.83 were defined as differentially expressed proteins (DEPs).

### 2.5. Bioinformatics Analysis

Principal component analysis (PCA) and Pearson correlation analysis were conducted in R v.4.4.1 to assess sample relationships and replicate consistency. DEGs and DEPs were annotated to the Kyoto Encyclopedia of Genes and Genomes (KEGG) and Gene Ontology (GO) databases to identify associated biological functions and pathways. Enrichment analysis was performed using a significance threshold of *p* < 0.05. In order to identify hub proteins with high connectivity, all DEGs and DEPs were mapped to STRING v.12.0 [31] to construct protein–protein interaction (PPI) networks. The PPI network was constructed using a combined interaction score > 900. Key DEGs and DEPs associated with major pathways were integrated to construct an mRNA–pathway–protein regulatory network. Network visualization was performed using Cytoscape v.3.10.1 [32].

### 2.6. Statistical Analysis

All statistical analyses were conducted using SPSS v.22.0 (SPSS Inc., Chicago, IL, USA). For comparisons between two groups, Student’s *t*-test was used. For comparisons among three or more groups, one-way analysis of variance (ANOVA) followed by Duncan’s multiple range test was applied. Each treatment was carried out in at least three replicates. Results are expressed as mean ± standard deviation (SD), and significance was defined as *p* < 0.05. Graphical representations were generated using GraphPad Prism v.9.5.0 (GraphPad Software, San Diego, CA, USA).

## 3. Results

### 3.1. Physiological Analysis

#### 3.1.1. Effect of Osmolality on Sperm Motility

**Figure 2:**
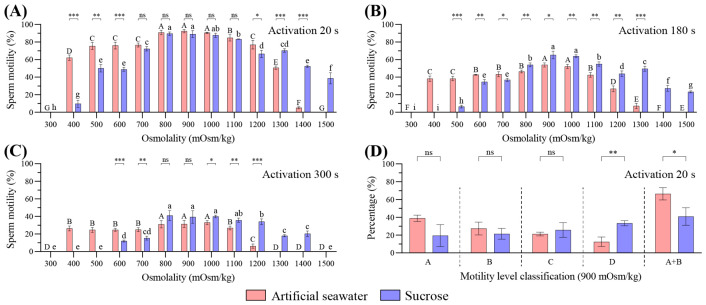
Effect of osmolality. Effects of artificial seawater and sucrose under varying osmotic conditions. Motility was measured at 20 s (**A**), 180 s (**B**), and 300 s (**C**) post-activation across 300–1500 mOsm/kg. Uppercase and lowercase letters indicate significant differences among osmolalities within artificial seawater and sucrose treatments, respectively (*p* < 0.05). Statistical significance is indicated as follows: ns, *p* > 0.05; *, *p* < 0.05; **, *p* < 0.01; ***, *p* < 0.001. (**D**) Motility level classification comparing treatments at 900 mOsm/kg and 20 s post-activation, including grades A, B, C, and D.

Sperm showed good activation (motility > 70%) within 500–1200 mOsm/kg in artificial seawater and 800–1100 mOsm/kg in sucrose (Figure 2A). At 20 s post-activation, high motility was maintained at 800–1000 mOsm/kg in both artificial seawater and sucrose (Figure 2A). At 180 and 300 s, optimal motility clustered around 900–1000 mOsm/kg (Figure 2B,C). Throughout all stages of activation, sperm motility was superior in artificial seawater compared to sucrose under low osmotic conditions. With increasing osmolarity, sucrose-mediated activation effect progressively approximated that of artificial seawater, and at elevated osmolarities, sucrose surpassed artificial seawater in promoting motility (Figure 2A–C). At 900 mOsm/kg, artificial seawater significantly increased the proportion of rapidly motile (A + B level) sperm and decreased weakly motile (D level) sperm at 20 s post-activation (*p* < 0.05) (Figure 2D). These results suggested that sperm activation and movement are primarily driven by osmolality, with ions exerting a modulatory influence.

#### 3.1.2. Effect of Ions on Sperm Motility

**Figure 3:**
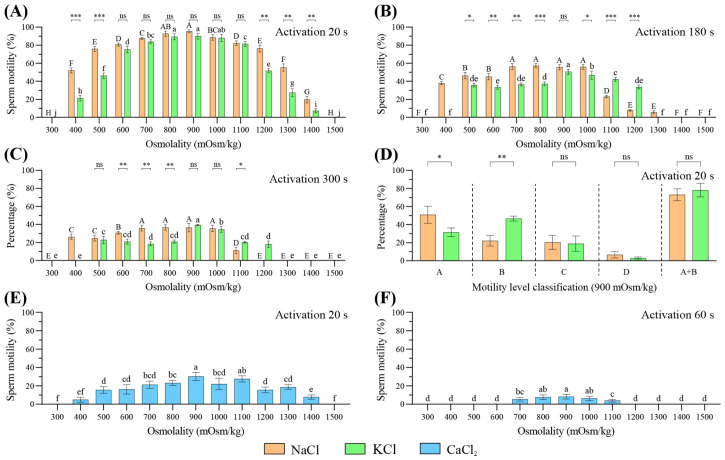
Effect of ions. Motility was measured at 20 s (**A**), 180 s (**B**), and 300 s (**C**) post-activation across different osmolalities in NaCl and KCl treatments. Different uppercase and lowercase letters indicate significant differences among osmolalities within NaCl and KCl treatments, respectively (*p* < 0.05). Statistical significance is indicated as follows: ns, *p* > 0.05; *, *p* < 0.05; **, *p* < 0.01; ***, *p* < 0.001. (**D**) Motility level classification at 900 mOsm/kg and 20 s post-activation in NaCl and KCl solutions, including grades A, B, C, and D. Motility was assessed at 20 s (**E**) and 60 s (**F**) post-activation in CaCl_2_ activation solution across a range of osmolalities. Lowercase letters indicate significant differences among treatments (*p* < 0.05).

The effects of Na^+^, K^+^, and Ca^2+^ on sperm motility were further examined. At 20 s post-activation, NaCl supported high motility at 800–900 mOsm/kg, whereas KCl maintained high motility at 800–1000 mOsm/kg (Figure 3A). At later time points, NaCl sustained high motility across 700–1000 mOsm/kg, while motility supported by KCl was confined to 900 mOsm/kg (Figure 3B,C). Under identical osmotic conditions, NaCl promoted higher motility than KCl at 400–500 and 1200–1400 mOsm/kg at 20 s, 500–800 mOsm/kg at 180 s, and 600–800 mOsm/kg at 300 s (*p* < 0.05) (Figure 3A–C). At 900 mOsm/kg and 20 s post-activation, NaCl significantly increased the proportion of rapidly motile (A level) sperm relative to KCl (*p* < 0.05) (Figure 3D). Notably, CaCl_2_ activation resulted in substantially reduced motility at both 20 s and 60 s (Figure 3E,F), suggesting a pronounced inhibitory effect of Ca^2+^ on sperm activation and movement.

#### 3.1.3. Effect of Combined Ions and pH on Sperm Motility

Given the consistently high motility observed at 900 mOsm/kg across artificial seawater, sucrose, NaCl, and KCl solutions, this osmolality was selected for combined ions analysis. At 900 mOsm/kg, overall sperm motility did not significantly differ among NaCl:KCl ratios (Figure S1A). Motility classification analysis showed that certain ion ratios improved sperm quality. At 20 s post-activation, NaCl:KCl ratios of 5:1, 3:1, and 1:3 significantly increased the proportion of A level sperm, and ratios of 3:1, 1:1, 1:3, and 1:7 significantly increased the proportion of A + B level sperm compared with other ratios (*p* < 0.05) (Figure S1B), suggesting a synergistic interaction between Na^+^ and K^+^ after sperm activation.

Using NaCl activation solution at 20 s post-activation and 900 mOsm/kg, sperm exhibited the highest motility at pH 7–9 (Figure S2A). A pH interval of 6–11 was further examined for motility level classification, showing that pH 8 significantly increased the proportion of rapidly motile sperm (A and A + B levels) and reduced weakly motile sperm (C and D levels) (Figure S2B). These results indicated that a slightly alkaline environment optimally supported sperm activation and movement.

### 3.2. Transcriptomics Analysis

#### 3.2.1. RNA-Seq Summary, Identification of DEGs, and PPI Network

**Figure 4:**
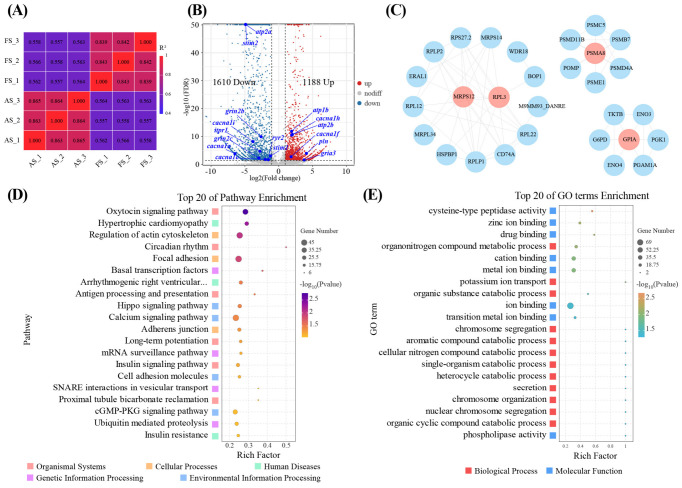
Transcriptomics analysis. (**A**) Pearson correlation coefficients among six sperm samples. (**B**) Volcano plot illustrating differentially expressed genes (DEGs) between activated sperm (AS) and fresh sperm (FS). (**C**) Protein–protein interaction (PPI) network constructed from DEGs. Red ellipses denote hub genes with high connectivity, and blue ellipses indicate interacting partners. (**D**) KEGG pathway enrichment analysis. (**E**) GO enrichment analysis.

As summarized in Table S1, RNA-seq generated 281,677,348 raw reads from six sperm samples. After quality filtering, 269,468,844 clean reads (95.67%) were retained, with clean bases ranging from 6.30 to 7.14 Gbp. GC content ranged from 49.01% to 49.93%, and Q20 and Q30 values exceeded 98% and 95%, respectively, indicating high sequencing quality. Mapping rates to the *L. maculatus* reference genome ranged from 27.93% to 33.22%, with uniquely mapped reads ranging from 25.19% to 29.74%. Transcriptome assembly identified 14,096 genes, including 11,624 known and 2472 novel genes. Pearson correlation coefficients demonstrated strong reproducibility among biological replicates within FS (0.839–0.843) and AS (0.863–0.865) groups, respectively (Figure 4A). A total of 2798 DEGs were identified between AS and FS, including 1610 up-regulated and 1188 down-regulated genes (Figure 4B; Supplementary Data S1). Several ion channel associated genes were differentially expressed, including *atp1b*, *gria3*, *ryr2*, *itpr1*, *pln*, *stim1*, *stim2*, *atp2a*, *atp2b*, *grin2b*, *grin2c*, *cacna1c*, *cacna1a*, *cacna1i*, *cacna1f*, and *cacna1h*. PPI network analysis further identified four hub genes (MRPS12, RPL3, PSMA8, and GPIA) (Figure 4C).

#### 3.2.2. KEGG and GO Enrichment Analyses of DEGs

KEGG enrichment analysis revealed 11 significantly enriched pathways (*p* < 0.05) in the AS vs. FS comparison (Figure 4D; Supplementary Data S1). These pathways included organismal system pathways, such as endocrine system, environmental adaptation, and immune system, as well as environmental information processing pathways, including signal transduction. Up-regulated DEGs were significantly enriched in circadian rhythm and insulin signaling pathway (Figure S3A), whereas down-regulated DEGs were significantly enriched in calcium signaling pathway (Figure S3B). These results suggested that signal transduction plays a prominent role in sperm activation and movement.

GO enrichment analysis identified 20 significantly enriched GO terms (*p* < 0.05), comprising 12 biological process (BP) and 8 molecular function (MF) categories (Figure 4E; Supplementary Data S1). Up-regulated DEGs were significantly enriched in organonitrogen compound metabolic process (GO:1901564) and transition metal ion binding (GO:0046914) (Figure S3C), whereas down-regulated DEGs were significantly enriched in calcium ion binding (GO:0005509) (Figure S3D). These findings reinforce the importance of ion signaling and metabolic processes after sperm activation.

### 3.3. Proteomics Analysis

#### 3.3.1. LC-MS/MS Summary, Identification of DEPs, and PPI Network

**Figure 5:**
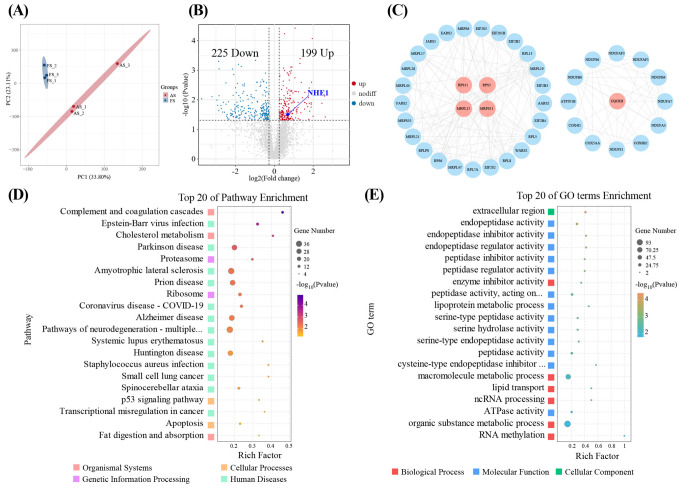
Proteomics analysis. (**A**) Principal component analysis (PCA) of six sperm samples. (**B**) Volcano plot illustrating differentially expressed proteins (DEPs) between AS and FS groups. (**C**) PPI network constructed from DEPs. Red ellipses denote hub proteins with high connectivity, and blue ellipses indicate interacting partners. (**D**) KEGG pathway enrichment analysis. (**E**) GO enrichment analysis.

Proteomic analysis identified 3939 proteins from 35,135 peptides. Functional annotation was performed against Clusters of Orthologous Groups of proteins (KOG/COG), KEGG, GO, Trans_Factor, InterPro (IPR), and Subcellular databases, with 65 proteins annotated across all datasets. PCA revealed clear separation between AS and FS groups (Figure 5A), indicating distinct proteomic signatures. A total of 424 DEPs were identified between AS and FS groups, including 199 up-regulated and 225 down-regulated proteins (Figure 5B; Supplementary Data S2). Ion channel-related protein (NHE1) was differentially expressed. PPI network analysis further identified five hub proteins, including RPS11, RPS5, MRPL15, MRPS11, and UQCRH (Figure 5C).

#### 3.3.2. KEGG and GO Enrichment Analyses of DEPs

KEGG pathway analysis revealed 20 significantly enriched pathways (*p* < 0.05) in the AS vs. FS comparison (Figure 5D; Supplementary Data S2). These pathways were primarily associated with the immune system, digestive system, folding, sorting and degradation, translation, and cell growth and death. Up-regulated DEPs were significantly enriched in proteasome, ribosome, and amino sugar and nucleotide sugar metabolism (Figure S4A), while down-regulated DEPs were significantly enriched in cholesterol metabolism, apoptosis, p53 signaling pathway, and PPAR signaling pathway (Figure S4B). These results indicated that translation, metabolism and apoptosis-related pathways contribute to sperm activation and movement.

GO enrichment analysis identified 40 significantly enriched GO terms (*p* < 0.05), including 22 BP, 1 cellular component (CC), and 17 MF (Figure 5E; Supplementary Data S2). Up-regulated DEPs were significantly enriched in cellular macromolecule metabolic process (GO:0044260), ATPase activity (GO:0016887), and cellular metabolic process (GO:0044237) (Figure S4C), whereas down-regulated DEPs were significantly enriched in cation transport (GO:0006812) and ion transmembrane transporter activity (GO:0015075) (Figure S4D). These findings highlight the metabolic processes and ion transporters as key features of sperm activation and movement.

### 3.4. Integrated Transcriptomics and Proteomics Reveal Sperm Activation and Movement Mechanisms

**Figure 6:**
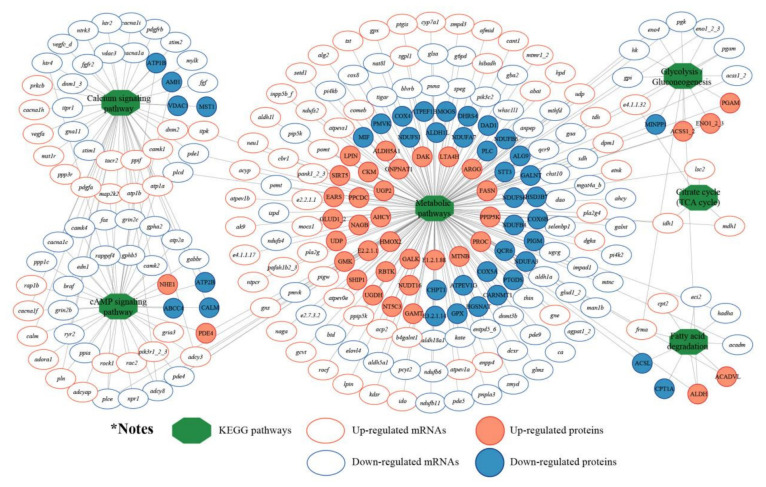
mRNA–pathway–protein network analysis. The pathway-DEGs-DEPs network included 6 key KEGG pathways, 187 DEGs, and 77 DEPs.

A total of 6 key KEGG pathways, 187 DEGs, and 77 DEPs were used to construct an mRNA–pathway–protein network (Figure 6). Integrative analysis of DEGs and DEPs uncovered coordinated alterations across multiple KEGG pathways, such as *pln* and *ryr2*, which were involved in calcium signaling pathways and cAMP signaling pathways.

**Figure 7:**
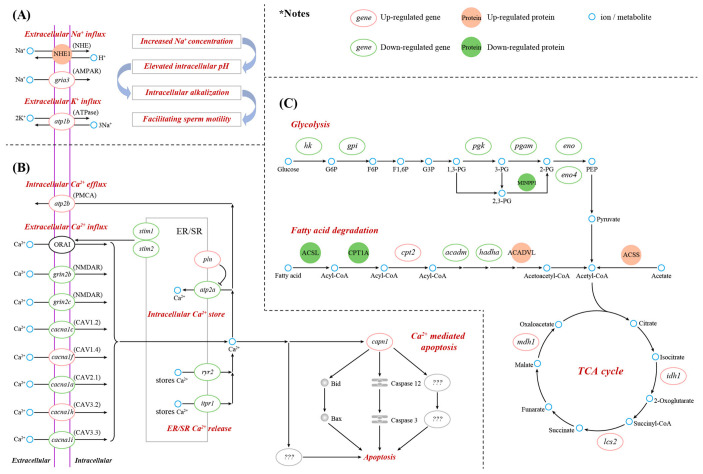
Transcriptomic and proteomic regulatory networks of spotted seabass after sperm activation related to (**A**) Na^+^ and K^+^ signaling, (**B**) Ca^2+^ signaling and apoptosis, and (**C**) energy metabolism.

Based on transcriptomic and proteomic analyses, we identified 29 genes and 6 proteins as key molecules that were related to Na^+^ and K^+^ signaling, Ca^2+^ signaling and apoptosis, as well as energy metabolism, all of which play critical roles in sperm activation and movement of spotted seabass. After sperm activation, genes associated with extracellular Na^+^ influx (*gria3*), extracellular K^+^ influx (*atp1b*), intracellular Ca^2+^ store regulator (*pln*), intracellular Ca^2+^ efflux (*atp2b*), extracellular Ca^2+^ influx (*cacna1f*, *cacna1h*), Ca^2+^-mediated apoptosis (*capn1*), TCA cycle (*idh1*, *lcs2*, *mdh1*), and fatty acid degradation (*cpt2*) were up-regulated, while genes related to intracellular Ca^2+^ stores (*atp2a*), ER/SR Ca^2+^ release (*ryr2*, *itpr1*), extracellular Ca^2+^ influx regulator (*stim1*, *stim2*), extracellular Ca^2+^ influx (*grin2b*, *grin2c*, *cacna1c*, *cacna1a*, *cacna1i*), glycolysis (*hk*, *gpi*, *pgk*, *pgam*, *eno*, *eno4*), and fatty acid degradation (*acadm*, *hadha*) were down-regulated (Table S2). At the protein expression level, proteins involved in extracellular Na^+^ influx (NHE1), glycolysis (ACSS), and fatty acid degradation (ACADVL) were up-regulated, while proteins associated with glycolysis (MINPP1) and fatty acid degradation (ACSL, CPT1A) were down-regulated (Table S3). Based on these results, a putative transcriptomic and proteomic regulatory network was constructed to elucidate the molecular mechanisms underlying sperm activation and movement in spotted seabass (Figure 7).

## 4. Discussion

### 4.1. Integrated Roles of Osmotic Effects and Ionic Regulation in Sperm Activation and Movement

In teleosts, osmolality plays a decisive role in sperm activation [7]. Spermatozoa are immotile in seminal plasma and initiate motility only after exposure to osmotic shifts, specifically hyperosmotic seawater in marine fishes or hypoosmotic freshwater in freshwater species [7,33]. Freshwater fish sperm are optimally activated under hypo-osmotic conditions across 60–140 mOsm/kg [9,34,35], whereas in marine fishes, activation occurs most effectively at 700–1000 mOsm/kg [36,37,38]. In our study, spotted seabass sperm maintained strong motility across 500–1200 mOsm/kg, exceeding the osmolality of seminal plasma (316–322 mOsm/kg), indicative of a broad activation range in this euryhaline fish. Similar broad osmotic activation ranges have been reported in several euryhaline teleosts, including Nile tilapia (*Oreochromis niloticus*, 0–150 mOsm/kg) [19], Japanese medaka (*Oryzias latipes*, 25–315 mOsm/kg) [39], Mozambique tilapia (*Oreochromis mossambicus*, 70–271 mOsm/kg) [40], Gulf killifish (*Fundulus grandis*, 400–800 mOsm/kg) [41], and black-chinned tilapia (*Sarotherodon melanotheron heudelotii*, 500–1000 mOsm/kg) [42]. However, the optimal osmotic range differs substantially among these species, indicating that osmotic regulation of sperm activation in euryhaline fishes is species-specific. These findings underscore the importance of broad osmotic adaptability in sperm physiology for euryhaline species.

Multi-ionic activation media produced higher sperm motility and increased the proportion of rapidly motile sperm in spotted seabass compared with the ion-free sucrose medium, highlighting the critical role of ionic composition in sperm activation. Our physiological results showed that NaCl supported strong motility across 700–1000 mOsm/kg, with optimal performance at 800–900 mOsm/kg and a higher proportion of rapidly motile sperm. One potential mechanism involves Na^+^-dependent activation of sperm-specific Na^+^/H^+^ exchanger (sNHE), which mediates extracellular Na^+^ influx and promotes the extrusion of intracellular H^+^, thereby increasing cytoplasmic pH [43,44,45]. Intracellular alkalization has been shown to stimulate dynein-driven flagellar beating and improve sperm motility through increased dynein ATPase activity [46]. The Na^+^ electrochemical gradient further contributes to membrane potential formation, which may modulate voltage-dependent K^+^ channels involved in the regulation of sperm motility [47,48]. In freshwater fishes, NaCl enhances sperm motility at 30–70 mM [9,34,35], whereas marine species require higher concentrations across 350–500 mM for optimal activation [36,37,38]. In euryhaline fishes, Na^+^ similarly exerts stimulatory effects on sperm motility, as reported in Mozambique tilapia [40], Japanese medaka [39], Gulf killifish [41], black-chinned tilapia [42], Nile tilapia [19], and spotted seabass. Overall, Na^+^ appears to have a generally stimulatory effect on sperm motility in many reported marine, freshwater, and euryhaline fishes, although the effective concentration range and regulatory context vary among species.

**Figure 8:**
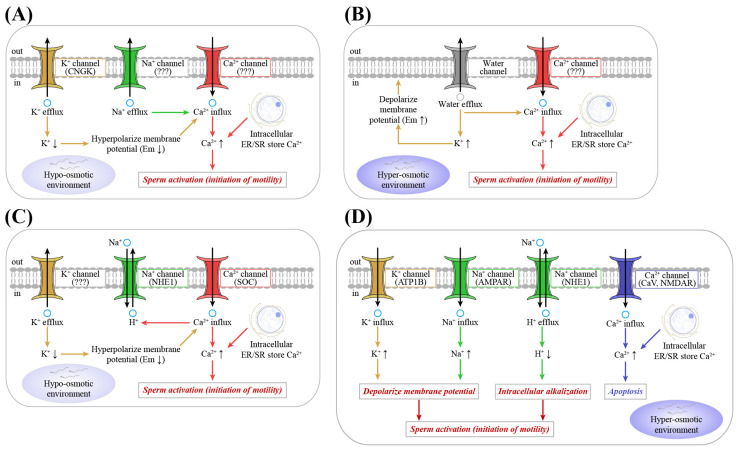
Models of sperm signaling pathways in most freshwater fishes [4], marine fishes [4], euryhaline Nile tilapia [19], and euryhaline spotted seabass (this study). (**A**) In most freshwater fishes, hypo-osmotic environment triggers K^+^ efflux, hyperpolarizing the membrane potential and promoting extracellular Ca^2+^ influx and Ca^2+^ release from intracellular store, thereby activating Ca^2+^-dependent signaling for sperm motility. (**B**) In marine fishes, hyper-osmotic environment induces intracellular water efflux and elevated K^+^, depolarizing membrane potential and enhancing fluidity, and extracellular Ca^2+^ influx triggers signaling that initiates flagellar beating and enhances motility. (**C**) In euryhaline Nile tilapia, under hypo-osmotic conditions, K^+^ efflux induces hyperpolarization and Ca^2+^ influx, activating signaling and Na^+^/H^+^ exchanger NHE1-mediated alkalization to enhance sperm motility. (**D**) In euryhaline spotted seabass, under hyper-osmotic conditions, Na^+^ and K^+^ influx depolarize membranes and promote membrane fluidity, and Na^+^/H^+^ exchanger NHE1-driven alkalization enhances motility; extracellular Ca^2+^ influx, however, suppresses motility and triggers apoptosis.

However, K^+^ exerts dual effects, blocking sperm motility in certain freshwater fishes but driving membrane depolarization and motility initiation in marine species. Freshwater fish sperm typically harbor higher intracellular K^+^ than the external environment [49,50]. Hypo-osmotic environment induces K^+^ efflux via CNGK channels, driven by a decrease in intracellular K^+^, leading to membrane hyperpolarization and signal cascades (Figure 8A) [4]. High K^+^ concentrations in activation media, therefore, inhibit sperm activation and motility in some freshwater fishes [7,8]. In hyper-osmotic environments, marine fish sperm experience water efflux, resulting in elevated intracellular K^+^ and membrane depolarization, thereby promoting sperm motility (Figure 8B) [4]. In this study, K^+^ supplemented activation solution promoted sperm activation in euryhaline spotted seabass, consistent with the stimulatory effect of K^+^ reported in euryhaline rainbow trout [11], but contrasting with the inhibitory effect of K^+^ activation media observed in euryhaline marbled goby [9] and Nile tilapia (Figure 8C) [19]. These findings suggested that euryhaline species with freshwater or seawater adaptive traits exhibit distinct regulatory responses to K^+^ after sperm activation. Additionally, our results indicated that under hyper-osmotic conditions, extracellular K^+^ may enter sperm via K^+^ channels, leading to increased intracellular K^+^ concentration and membrane depolarization, and thereby enhancing sperm activation and motility in euryhaline spotted seabass (Figure 8D).

Notably, Ca^2+^-mediated signaling is typically crucial for sperm activation and motility in most teleosts, yet in some species it functions as an inhibitory signal. In most freshwater fishes, hypo-osmotic conditions provoke K^+^ efflux and membrane hyperpolarization, resulting in Ca^2+^ influx through membrane channels that activates flagellar Ca^2+^/calmodulin-dependent proteins to initiate and sustain sperm motility (Figure 8A) [4]. By contrast, in marine fishes, hyper-osmotic response causes sperm shrinkage and membrane depolarization, mobilizing Ca^2+^ release from intracellular stores to activate Ca^2+^ signaling and drive axonemal beating (Figure 8B) [4]. Our results showed that CaCl_2_ activation media markedly suppressed sperm motility of spotted seabass, indicating that Ca^2+^ is not required for motility initiation in this euryhaline species. Similar findings have also been reported in some freshwater fishes, such as Java carp (*Puntius javanicus*) [9] and bluegill (*Lepomis macrochirus*) [35], in which the addition of Ca^2+^ to the activation solution inhibits sperm motility. Conversely, activation solutions supplemented with Ca^2+^ promote sperm activation and improve motility in euryhaline marbled goby [9] and Nile tilapia (Figure 8C) [19]. These findings reveal that Ca^2+^, similar to K^+^, regulates sperm activation and movement differently in euryhaline species, depending on their freshwater or seawater adaptive strategies.

### 4.2. Transcriptomics and Proteomics Reveal Activation and Movement Regulatory Networks

In this study, we used transcriptomics and proteomics to reveal activation and movement regulatory networks. Although the RNA-seq mapping rates were lower than those commonly observed in somatic tissue transcriptomes, this likely reflects the distinctive RNA composition of mature spermatozoa. These cells contain limited, fragmented, sperm-specific, and non-canonical RNA populations, many of which may be incompletely represented in the present *L. maculatus* genome annotation. Residual unannotated transcripts, incomplete sperm-specific annotation, and potential technical factors may also have contributed to the reduced mapping efficiency. Despite this limitation, the RNA-seq libraries showed high sequence quality and good reproducibility among biological replicates. Proteomic profiles further showed strong consistency within groups and clear separation between FS and AS groups. Moreover, despite the limited transcriptional activity of mature spermatozoa, these data provided useful clues for identifying candidate pathways associated with sperm movement. Accordingly, we integrated physiological results, transcriptomics, and proteomics to establish a putative regulatory framework for sperm activation and movement.

#### 4.2.1. Na^+^ and K^+^ Signaling

Integrated transcriptomic and proteomic analyses revealed a coordinated up-regulation of the *gria3* gene and NHE1 protein expression, indicating that Na^+^-associated ion transport mechanisms may be involved in sperm activation and movement in spotted seabass. The *gria3* gene encodes the GluA3 subunit of AMPA-type glutamate receptors (AMPARs), ligand-gated cation channels that open upon glutamate binding to mediate rapid Na^+^ influx across the plasma membrane [51]. Because AMPARs exhibit limited selectivity among monovalent cations, Na^+^ permeates through the pore, generating membrane depolarization that can regulate downstream ion channels and intracellular signaling cascades [51,52]. Solute carrier family 9 member 1 (SLC9A1, NHE1) is a widely expressed plasma membrane Na^+^/H^+^ exchanger that regulates intracellular Na^+^ levels and pH, thereby promoting intracellular alkalization that facilitates sperm motility [46,53]. Thus, up-regulated expression of Na^+^-associated genes and proteins suggested that Na^+^-related ion channels may facilitate extracellular Na^+^ influx, intracellular alkalization, and activation of downstream signaling cascades, ultimately triggering sperm activation and enhancing motility (Figure 7A). A similar Na^+^/H^+^ exchanger has also been identified in sea urchin sperm [43,54], supporting a potential involvement of NHE1 in regulating intracellular pH and motility.

Furthermore, our transcriptomic data showed that *atp1b*, encoding the Na^+^/K^+^-transporting ATPase subunit beta [55], was significantly up-regulated after sperm activation. Extracellular K^+^ influx through Na^+^/K^+^-ATPase and K^+^-selective channels depolarizes sperm membranes, activating cascades that drive flagellar beating [56,57]. The *atp1a*/*atp1b*-mediated K^+^ gradients are similarly essential in mammalian sperm for motility initiation and hyperactivation [58,59]. Up-regulation of *atp1b* expression may reflect an increased capacity for Na^+^/K^+^ transport, promoting sperm motility and flagellar beating (Figure 7A).

#### 4.2.2. Ca^2+^ Signaling and Apoptosis

It is noteworthy that genes involved in Ca^2+^ transport, including store Ca^2+^ release, intracellular Ca^2+^ storage and efflux, and extracellular Ca^2+^ influx, showed significant transcriptional changes after sperm activation (Figure 7B). In the present study, *ryr2* and *itpr1*, which mediate Ca^2+^ release from endoplasmic/sarco (ER/SR)-like intracellular stores [60,61], were significantly down-regulated, indicating a potential reduction in Ca^2+^ mobilization from intracellular stores. Phospholamban (PLN) negatively regulates the ER/SR Ca^2+^-ATPase (ATP2A/SERCA) by directly interacting with the pump and reducing its Ca^2+^ transport activity [62,63]. Plasma membrane Ca^2+^-ATPase (ATP2B/PMCA) acts as a key Ca^2+^ efflux pump that maintains intracellular Ca^2+^ homeostasis, and its activity is essential for regulating sperm motility [64,65]. The up-regulation of *pln* and *atp2b*, together with the down-regulation of *atp2a*, may reflect reduced intracellular Ca^2+^ uptake into ER/SR stores and increased Ca^2+^ extrusion from sperm cells.

ER Ca^2+^ store depletion activates the Ca^2+^ sensor, stromal interaction molecules (STIM1/2), thereby activating calcium channel (ORAI)-mediated store-operated Ca^2+^ channels (SOC) to promote extracellular Ca^2+^ influx [66,67]. N-methyl-D-aspartate receptors (NMDARs) are ligand-gated ion channels that allow extracellular Ca^2+^ influx upon agonist binding and membrane depolarization [68]. In this study, significant down-regulation of Ca^2+^ sensor-related genes (*stim1*, *stim2*) and NMDAR subunit genes (*grin2b*, *grin2c*), together with the inhibitory effects of Ca^2+^ observed in sperm motility assays, indicated that Ca^2+^ influx may not be required for sperm movement in spotted seabass. Voltage-dependent calcium channels (VDCCs) open in response to membrane depolarization and permit extracellular Ca^2+^ influx [69,70]. However, transcriptomic data revealed heterogeneous regulation of VDCC-related genes, with three down-regulated (*cacna1c*, *cacna1a*, *cacna1i*) and two up-regulated (*cacna1f*, *cacna1h*), suggesting that Ca^2+^ entry may be related to the maintenance of intracellular ionic homeostasis. Taken together, both physiology and multi-omics analyses indicated that extracellular Ca^2+^ is not required for sperm movement in spotted seabass and may negatively affect sperm motility.

In addition, our transcriptomic data identified a significant up-regulation of *capn1*, a gene encoding the Ca^2+^-activated protease calpain-1 [71], suggesting a possible involvement of Ca^2+^-dependent proteolytic pathways after sperm activation. Upon Ca^2+^ binding, calpain-1 can become activated and cleaves cytoskeletal and regulatory proteins (e.g., caspase-12), leading to the initiation of apoptotic signaling cascades and Ca^2+^-mediated cell death [71,72]. These findings suggested that Ca^2+^ induces rapid loss of sperm viability following activation, likely through calpain-1-mediated apoptosis (Figure 7B). Moreover, additional mechanisms underlying Ca^2+^-induced inhibition of sperm activation and movement remain to be elucidated. Collectively, our results indicated an inhibitory role of Ca^2+^ in sperm activation and movement, highlighting that reducing Ca^2+^ exposure will improve fertilization efficiency in aquaculture practices.

#### 4.2.3. Energy Metabolism

Fish sperm rely on tightly regulated energy metabolism to power motility, with ATP supply being essential for initiating and sustaining flagellar movement and ensuring fertilization success [17,73]. Prior to activation, ATP is primarily synthesized and stored via mitochondrial oxidative phosphorylation, while post-activation energy can be supplied by metabolic intermediates, concurrent with glycolytic suppression and a metabolic transition toward alternative energy pathways [74,75]. In this study, our transcriptomic data showed that TCA cycle-related genes (*idh1*, *lcs2*, *mdh1*) were significantly up-regulated, suggesting that oxidative phosphorylation-linked TCA cycle activity plays an important role in sperm movement of spotted seabass. Glycolysis-related genes (*hk*, *gpi*, *pgk*, *pgam*, *eno*) and the associated protein (MINPP1) were significantly down-regulated, indicating reduced glycolytic activity, which is similar to the expression patterns previously reported in euryhaline Nile tilapia [19]. However, previous studies in Nile tilapia showed that increased expression of ACSL and CPT1 enhances the conversion of fatty acids to acyl-CoA, facilitating fatty acid metabolism [19]. Our data revealed significant down-regulation of fatty acid degradation-related genes (*acadm*, *hadha*) and proteins (ACSL, CPT1A) in spotted seabass sperm, suggesting that fatty acid metabolism may not be driven by fatty acid synthesis followed by oxidation. Instead, the significant up-regulation of *cpt2* and ACADVL, a key enzyme initiating fatty acid oxidation of very-long-chain fatty acids [76], may indicate a potential contribution of very-long-chain fatty acid degradation to acetyl-CoA production and the TCA cycle.

Additionally, we detected a significant increase in ACSS protein abundance after sperm activation. ACSS catalyzes the ATP-dependent conversion of acetate into acetyl-CoA, thereby linking acetate metabolism with central carbon metabolism [77,78]. Therefore, up-regulation of ACSS suggested that acetate-derived acetyl-CoA production may provide substrates for the TCA cycle to sustain ATP generation required for sperm motility. Collectively, these findings indicated that oxidative metabolism through the TCA cycle may represent an important energy-related pathway supporting sperm movement in spotted seabass, potentially involving acetyl-CoA derived from very-long-chain fatty acid degradation and acetate utilization (Figure 7C).

### 4.3. Implications for Aquaculture, Limitations of Omics, and Prospects

Our physiological findings provide preliminary guidance for optimizing sperm activation media in spotted seabass. Activation media based on NaCl, KCl, or their mixtures, prepared at 26.3‰ salinity with a total concentration of 450 mM and an osmolality of approximately 900 mOsm/kg, effectively supported sperm activation and motility. In contrast, the inclusion of Ca^2+^ impaired sperm activation and motility, indicating that high concentration of Ca^2+^ should be excluded from activation media. Other divalent cations, such as Mn^2+^, Mg^2+^, and Sr^2+^, may also influence fish sperm activation and motility [35]. Our multi-omics analyses identified several candidate molecules and pathways linked to sperm activation and movement, but these results remain preliminary and correlative. Therefore, our results provided a putative model of the molecular mechanisms. Further studies using pharmacological inhibitor assays, Western blot analyses, electrophysiology, immunocytochemistry, and in situ hybridization [45,79] are required to validate the roles of these key molecules and pathways in sperm activation and motility in spotted seabass.

## 5. Conclusions

**Figure 9:**
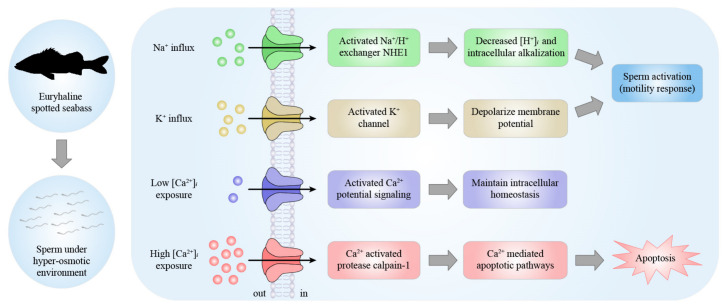
Physiological and molecular mechanisms underlying sperm activation and movement in euryhaline spotted seabass.

This study provides physiological and multi-omics insights into the putative mechanisms underlying sperm activation and movement in euryhaline spotted seabass (Figure 9). Spotted seabass sperm exhibited a broader osmotic activation range than freshwater and marine species, suggesting that enhanced osmotic adaptability may be a key feature of euryhaline fish sperm physiology. Na^+^ and K^+^ activation media markedly enhanced sperm motility, whereas the addition of Ca^2+^ suppressed sperm movement. Our transcriptomic and proteomic analyses elucidated regulatory networks underlying sperm activation and movement, in which key genes and proteins were mainly related to Na^+^ and K^+^ signaling, Ca^2+^ signaling and apoptosis, and energy metabolism. The increased abundance of NHE1 suggests a potential involvement of Na^+^ influx and intracellular alkalization, which may contribute to sperm motility. Activation of K^+^ channels triggered extracellular K^+^ influx, which may promote membrane depolarization and fluidity to drive flagellar beating. Notably, up-regulation of calpain-1 may be related to Ca^2+^-mediated apoptosis, underscoring the importance of minimizing Ca^2+^ exposure during fertilization and hatchery operations. In addition, sperm movement is likely primarily supported by oxidative phosphorylation via the TCA cycle, fueled by acetyl-CoA derived from very-long-chain fatty acid degradation and acetate utilization rather than glycolysis, which may be a critical energy adaptation strategy in euryhaline fish. Collectively, our findings emphasize the integrated roles of osmotic effects and ionic regulation, providing novel insights into sperm activation and movement mechanisms in euryhaline spotted seabass and offering a useful reference for studies of other euryhaline fishes.

## Data Availability

All transcriptomic and proteomic raw data are deposited in the NCBI Sequence Read Archive (SRA) database (accession number PRJNA1440895) and the National Genomics Data Center (NGDC, China) OMIX database (https://ngdc.cncb.ac.cn/, accessed on 2 July 2026, accession number OMIX015742), respectively.

## Supplementary Materials

The following supporting information can be downloaded at https://www.mdpi.com/article/10.3390/biom16071021/s1. Figure S1: Effect of combined ions on sperm motility; Figure S2: Effect of combined pH on sperm motility; Figure S3: Enrichment analysis of up-regulated and down-regulated DEGs; Figure S4: Enrichment analysis of up-regulated and down-regulated DEPs; Table S1: Summary of RNA-seq; Table S2: Key genes related to Na^+^ and K^+^ signaling, Ca^2+^ signaling and apoptosis, and energy metabolism; Table S3: Key proteins related to Na^+^ signaling and energy metabolism; Supplementary_Data_S1: Transcriptomics; Supplementary_Data_S2: Proteomics.

## Abbreviations

The following abbreviations are used in this manuscript:

*Acadm*	acyl-CoA dehydrogenase
*atp1b*	sodium/potassium-transporting ATPase subunit beta
*atp2a*	P-type Ca2+ transporter type 2A
*atp2b*	P-type Ca2+ transporter type 2B
*cacna1a*	voltage-dependent calcium channel P/Q type alpha-1A (CAV2.1)
*cacna1c*	voltage-dependent calcium channel L type alpha-1C (CAV1.2)
*cacna1f*	voltage-dependent calcium channel L type alpha-1F (CAV1.4)
*cacna1h*	voltage-dependent calcium channel T type alpha-1H (CAV3.2)
*cacna1i*	voltage-dependent calcium channel T type alpha-1I (CAV3.3)
*capn1*	calpain-1
*cpt2*	carnitine O-palmitoyltransferase 2
*Eno*	enolase
*eno4*	enolase 4
*gpi*	glucose-6-phosphate isomerase
*gria3*	glutamate receptor 3
*grin2b*	glutamate receptor ionotropic, NMDA 2B
*grin2c*	glutamate receptor ionotropic, NMDA 2C
*Hadha*	enoyl-CoA hydratase
*Hk*	hexokinase
*idh1*	isocitrate dehydrogenase
*itpr1*	inositol 1,4,5-triphosphate receptor type 1
*lcs2*	succinyl-CoA synthetase beta subunit
*mdh1*	malate dehydrogenase
*Pgam*	2,3-bisphosphoglycerate-dependent phosphoglycerate mutase
*Pgk*	phosphoglycerate kinase
*Pln*	phospholamban
*ryr2*	ryanodine receptor 2
*stim1*	stromal interaction molecule 1
*stim2*	stromal interaction molecule 2
ACADVL	very long chain acyl-CoA dehydrogenase
ACSL	long-chain acyl-CoA synthetase
ACSS	acetyl-CoA synthetase
CPT1A	carnitine O-palmitoyltransferase 1
MINPP1	inositol-polyphosphate phosphatase
NHE1	solute carrier family 9 (sodium/hydrogen exchanger), member 1
