# Supplementary material for: Physiology and Multi-Omics Provide Insights into Sperm Activation and Movement in Euryhaline Spotted Seabass (Lateolabrax maculatus)
Source: Biomolecules. 2026 Jul 13;16(7):1021. doi: 10.3390/biom16071021 (PMC13406637; doi:10.3390/biom16071021)
Supplement: Supplementary file 1 [file biomolecules-16-01021-s001.zip › Supplementary_Figures.pdf]

## Supplementary Figures

# Physiology and multi-omics provide insights into sperm activation and movement in euryhaline spotted seabass (*Lateolabrax maculatus*)

Qinghua Wang[1], Yuxin Zhang[1], Weiwei Zhang[1], Yingxin Wu[1], Jiajie Li[1], Yizheng Zhang[1], Lu Li[1], Zhiming Zhu[1, 2, *], Zining Meng[1, 2, *]

[1] School of Life Sciences, State Key Laboratory of Biocontrol / Guangdong Core Germplasm Bank for Marine Economic Animals, Southern Marine Science and Engineering Guangdong Laboratory (Zhuhai) / Guangdong Provincial Key Laboratory of Aquatic Economic Animals, Sun Yat-sen University, Guangzhou 510275, China

[2] China-ASEAN Belt and Road Joint Laboratory on Mariculture Technology, Guangzhou 510275, China

[*] Corresponding author. School of Life Sciences, Sun Yat-sen University, Guangzhou 510275, China.

E-mail address: zhuzhiming@sml-zhuhai.cn (Z. Zhu); mengzn@mail.sysu.edu.cn (Z. Meng).

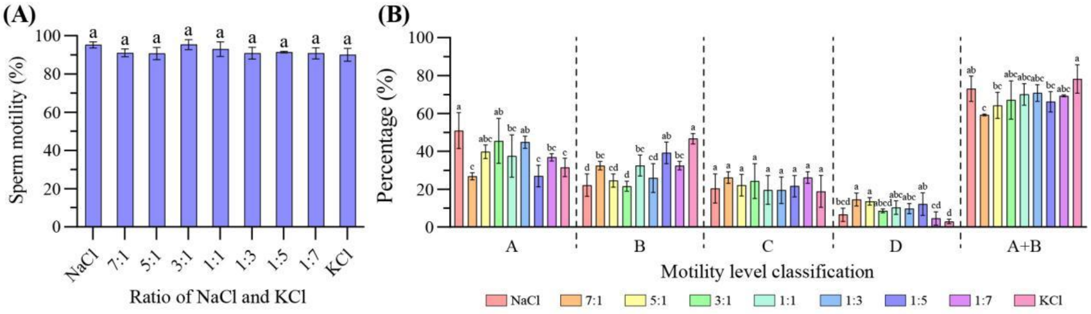


**Figure S1.** Effect of combined ions on sperm motility. (A) Sperm motility of spotted seabass at 900 mOsm/kg and 20 s post-activation under varying NaCl and KCl combinations. Different lowercase letters represent significant differences among treatments ($P < 0.05$). (B) Motility level classification (A, B, C, and D) under the same conditions. Different lowercase letters indicate significant differences within each motility category ($P < 0.05$).

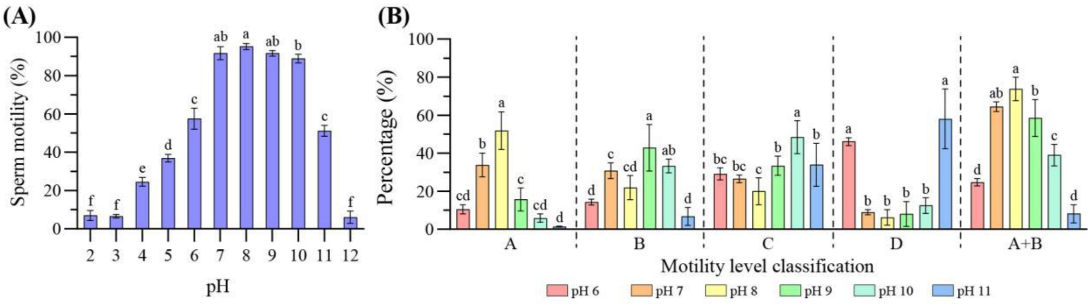


**Figure S2.** Effect of combined pH on sperm motility. (A) Sperm motility of spotted seabass at 900 mOsm/kg and 20 s post-activation using NaCl-based activation medium under varying pH. Different lowercase letters represent significant differences among treatments ($P < 0.05$). (B) Motility level classification (A, B, C, and D) under the same conditions. Different lowercase letters indicate significant differences within each motility category ($P < 0.05$).

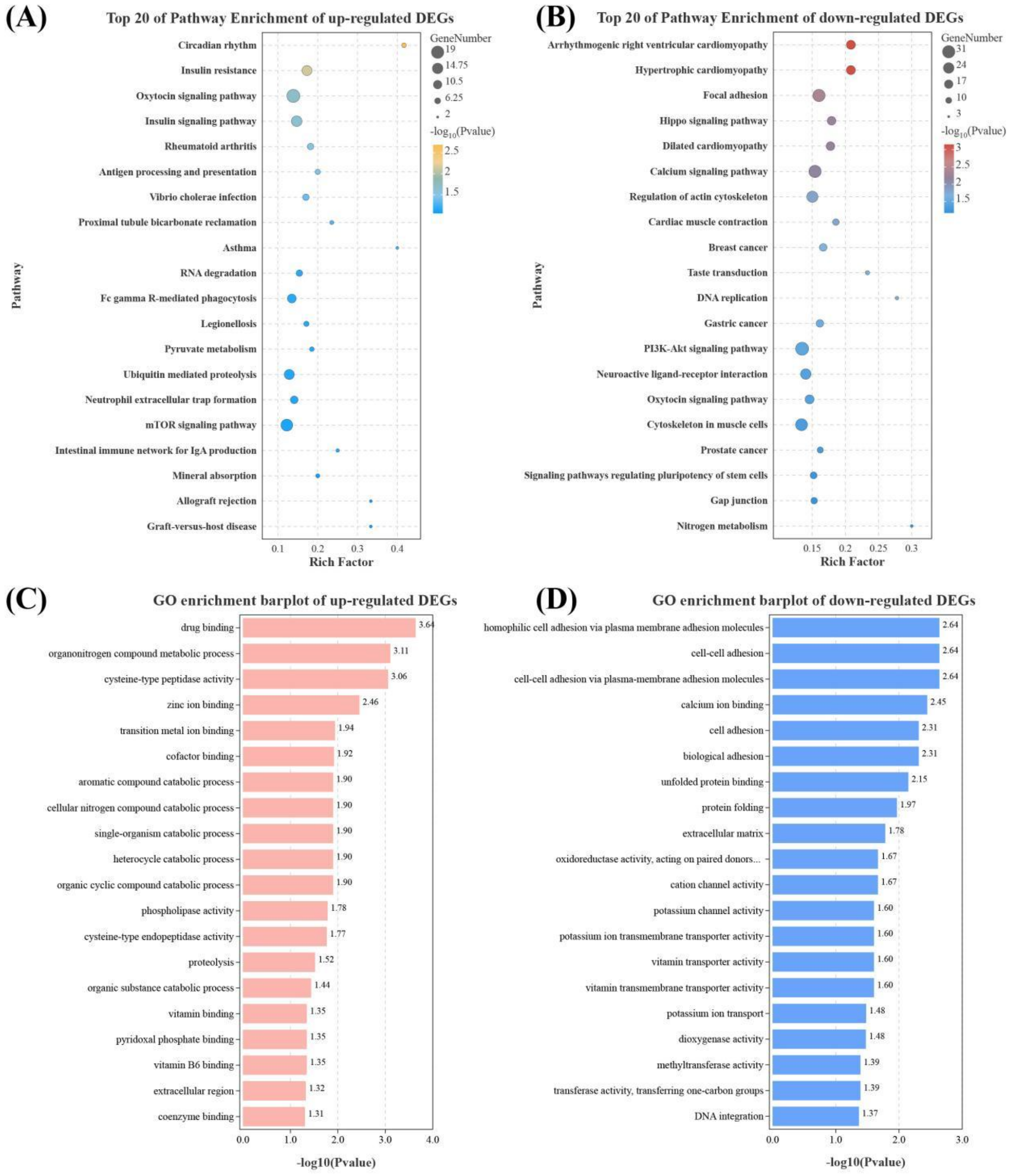


**Figure S3.** Enrichment analysis of up-regulated and down-regulated DEGs. KEGG enrichment analysis in AS vs. FS comparison of up-regulated (A) and down-regulated (B) DEGs. GO enrichment analysis in AS vs. FS comparison of up-regulated (C) and down-regulated (D) DEGs.

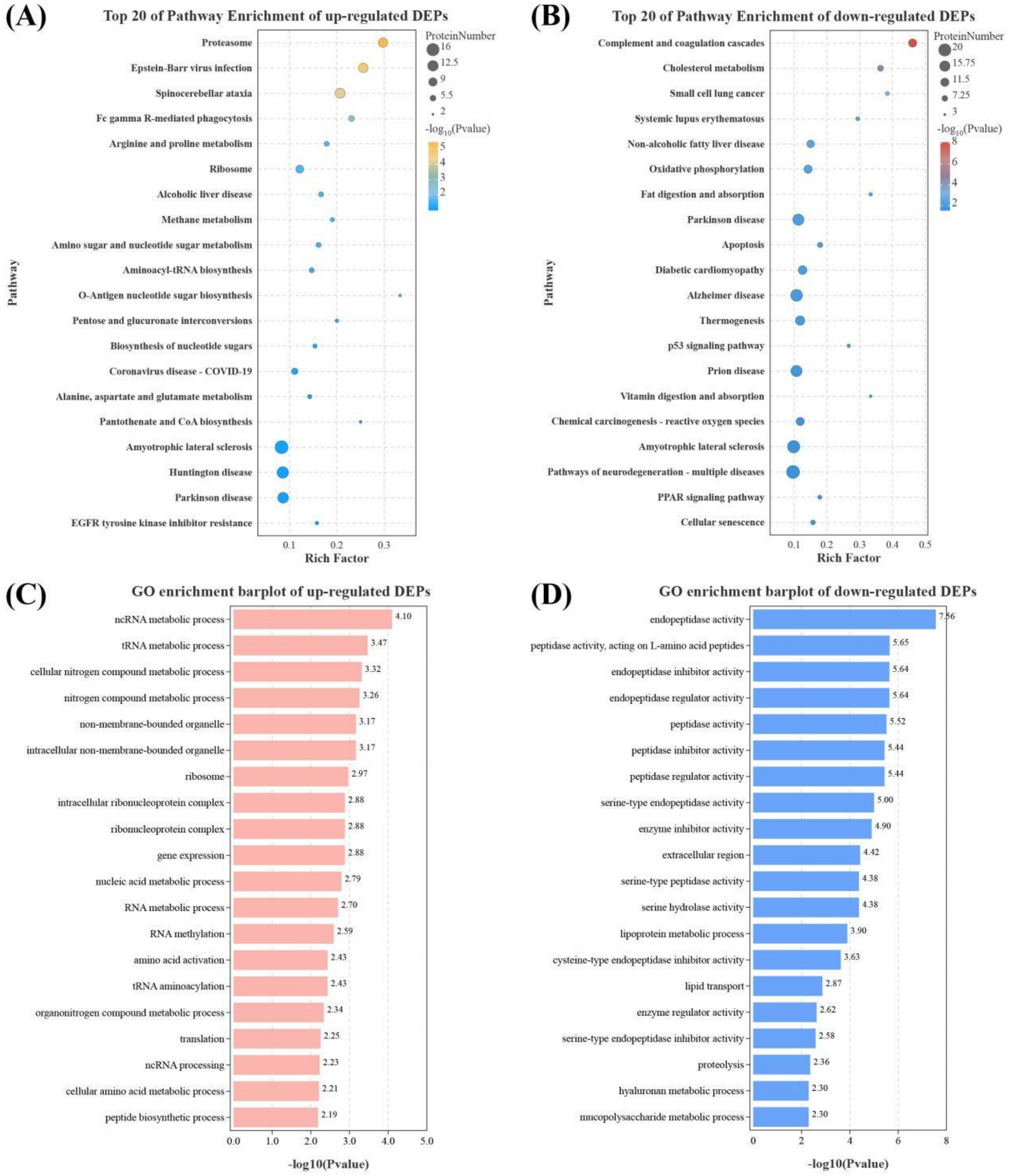


**Figure S4.** Enrichment analysis of up-regulated and down-regulated DEPs. KEGG enrichment analysis in AS vs. FS comparison of up-regulated (A) and down-regulated (B) DEPs. GO enrichment analysis in AS vs. FS comparison of up-regulated (C) and down-regulated (D) DEPs.
