# Supplementary material for: Physiology and Multi-Omics Provide Insights into Sperm Activation and Movement in Euryhaline Spotted Seabass (Lateolabrax maculatus)
Source: Biomolecules. 2026 Jul 13;16(7):1021. doi: 10.3390/biom16071021 (PMC13406637; doi:10.3390/biom16071021)
Supplement: Supplementary file 1 [file biomolecules-16-01021-s001.zip › Supplementary_Tables.pdf]

Supplementary Tables

Physiology and multi-omics provide insights into sperm activation and movement in euryhaline spotted seabass (*Lateolabrax maculatus*)

Qinghua Wang[1], Yuxin Zhang[1], Weiwei Zhang[1], Yingxin Wu[1], Jiajie Li[1], Yizheng Zhang[1], Lu Li[1], Zhiming Zhu[1, 2, *], Zining Meng[1, 2, *]

[1] School of Life Sciences, State Key Laboratory of Biocontrol / Guangdong Core Germplasm Bank for Marine Economic Animals, Southern Marine Science and Engineering Guangdong Laboratory (Zhuhai) / Guangdong Provincial Key Laboratory of Aquatic Economic Animals, Sun Yat-sen University, Guangzhou 510275, China

[2] China-ASEAN Belt and Road Joint Laboratory on Mariculture Technology, Guangzhou 510275, China

[*] Corresponding author. School of Life Sciences, Sun Yat-sen University, Guangzhou 510275, China.

E-mail address: zhuzhiming@sml-zhuhai.cn (Z. Zhu); mengzn@mail.sysu.edu.cn (Z. Meng).

Table S1. Summary of RNA-seq.

Sample	Raw reads	Clean reads	Raw bases (Gbp)	Clean bases (Gbp)	GC (%)	Q20 (%)	Q30 (%)	Total mapped (%)	Unique mapped (%)
FS_1	47,449,646	45,418,656	7.12	6.81	49.75	98.31	95.66	32.36	28.97
FS_2	46,197,304	42,022,272	6.93	6.3	49.93	98.01	95.11	31.28	27.91
FS_3	46,220,266	42,626,466	6.93	6.39	49.84	98.40	95.88	33.22	29.74
AS_1	46,783,864	46,081,798	7.02	6.91	49.27	98.45	95.85	28.94	26.29
AS_2	46,541,468	45,687,992	6.98	6.85	49.11	98.47	95.93	28.83	26.07
AS_3	48,484,800	47,631,660	7.27	7.14	49.01	98.45	95.91	27.93	25.19

Table S2. Key genes related to Na^+ and K^+ signaling, Ca^{2+} signaling and apoptosis, and energy metabolism.

Gene ID	Name	Description	Log2FC	Regulation	Biological process/pathway
**Na^+ and K^+, Ca^{2+} signaling**					
evm_model_ptg000002l_675	*gria3*	glutamate receptor 3	3.82	Up	Extracellular Na^+ influx
evm_model_ptg000001l_856	*atp1b*	sodium/potassium-transporting ATPase subunit beta	1.95	Up	Extracellular K^+ influx
**Ca^{2+} signaling and apoptosis**					
evm_model_ptg000020l_321	*pln*	phospholamban	4.15	Up	Intracellular Ca^{2+} store regulator
evm_model_ptg000009l_1187	*atp2a*	P-type Ca2+ transporter type 2A	-4.86	Down	Intracellular Ca^{2+} store
evm_model_ptg000005l_929	*ryr2*	ryanodine receptor 2	-1.49	Down	ER/SR Ca^{2+} release
evm_model_ptg000018l_1004	*itpr1*	inositol 1,4,5-triphosphate receptor type 1	-2.73	Down	ER/SR Ca^{2+} release
evm_model_ptg000007l_333	*stim1*	stromal interaction molecule 1	-1.16	Down	Extracellular Ca^{2+} influx regulator
evm_model_ptg000009l_607	*stim2*	stromal interaction molecule 2	-4.84	Down	Extracellular Ca^{2+} influx regulator
evm_model_ptg000016l_442	*atp2b*	P-type Ca2+ transporter type 2B	2.07	Up	Intracellular Ca^{2+} efflux
evm_model_ptg000009l_639	*grin2b*	glutamate receptor ionotropic, NMDA 2B	-2.56	Down	Extracellular Ca2+ influx
evm_model_ptg000021l_161	*grin2c*	glutamate receptor ionotropic, NMDA 2C	-2.89	Down	Extracellular Ca^{2+} influx
evm_model_ptg000016l_423	*cacna1c*	voltage-dependent calcium channel L type alpha-1C (CAV1.2)	-1.95	Down	Extracellular Ca^{2+} influx
evm_model_ptg000021l_729	*cacna1a*	voltage-dependent calcium channel P/Q type alpha-1A (CAV2.1)	-6.43	Down	Extracellular Ca^{2+} influx
evm_model_ptg000009l_219	*cacna1i*	voltage-dependent calcium channel T type alpha-1I (CAV3.3)	-3.76	Down	Extracellular Ca^{2+} influx
evm_model_ptg000006l_825	*cacna1f*	voltage-dependent calcium channel L type alpha-1F (CAV1.4)	1.89	Up	Extracellular Ca^{2+} influx
evm_model_ptg000004l_338	*cacna1h*	voltage-dependent calcium channel T type alpha-1H (CAV3.2)	1.97	Up	Extracellular Ca^{2+} influx
evm_model_ptg000014l_592	*capn1*	calpain-1	1.67	Up	Ca^{2+} mediated apoptosis
**Energy metabolism**					
evm_model_ptg000009l_168	*hk*	hexokinase	-2.03	Down	Glycolysis
evm_model_ptg000019l_1042	*gpi*	glucose-6-phosphate isomerase	-1.59	Down	Glycolysis
evm_model_ptg000002l_156	*pgk*	phosphoglycerate kinase	-2.61	Down	Glycolysis
evm_model_ptg000009l_862	*pgam*	2,3-bisphosphoglycerate-dependent phosphoglycerate mutase	-5.16	Down	Glycolysis
evm_model_ptg000012l_31	*eno*	enolase	-3.82	Down	Glycolysis
evm_model_ptg000014l_1388	*eno4*	enolase 4	-2.16	Down	Glycolysis
evm_model_ptg000013l_88	*idh1*	isocitrate dehydrogenase	2.02	Up	TCA cycle
evm_model_ptg000013l_45	*lcs2*	succinyl-CoA synthetase beta subunit	2.55	Up	TCA cycle
evm_model_ptg000014l_186	*mdh1*	malate dehydrogenase	2.74	Up	TCA cycle
evm_model_ptg000008l_209	*acadm*	acyl-CoA dehydrogenase	-5.68	Down	Fatty acid degradation
evm_model_ptg000010l_689	*hadha*	enoyl-CoA hydratase	-4.57	Down	Fatty acid degradation
novel.74	*cpt2*	carnitine O-palmitoyltransferase 2	3.85	Up	Fatty acid degradation

Table S3. Key proteins related to Na^+ signaling and energy metabolism.

Protein ID	Name	Description	FC	Regulation	Biological process/pathway
**Na^+ signaling**					
evm.model.ptg000004l.880	NHE1	solute carrier family 9 (sodium/hydrogen exchanger), member 1	1.62	Up	Extracellular Na^+ influx
**Energy metabolism**					
evm.model.ptg000014l.361	MINPP1	inositol-polyphosphate phosphatase	0.24	Down	Glycolysis
evm.model.ptg000006l.1040	ACSS	acetyl-CoA synthetase	1.48	Up	Glycolysis
evm.model.ptg000009l.496	ACSL	long-chain acyl-CoA synthetase	0.43	Down	Fatty acid degradation
evm.model.ptg000005l.550	CPT1A	carnitine O-palmitoyltransferase 1	0.61	Down	Fatty acid degradation
evm.model.ptg000012l.469	ACADVL	very long chain acyl-CoA dehydrogenase	2.20	Up	Fatty acid degradation
